# The Influence of Symptomatic Status on Post-endoscopic Retrograde Cholangiopancreatography (ERCP) Complications in Choledocholithiasis: A Systematic Review and Meta-Analysis

**DOI:** 10.7759/cureus.59322

**Published:** 2024-04-29

**Authors:** Akash Patel, Utsav P Vaghani, Sarang Mehta, Prijesh A Avaiya, Meet Virani, Fenilkumar Gorasiya

**Affiliations:** 1 Internal Medicine, Eisenhower Health, Rancho Mirage, USA; 2 Internal Medicine, Smt. Nathiba Hargovandas Lakhmichand (NHL) Municipal Medical College, Ahmedabad, IND; 3 Internal Medicine, Manila Central University-Filemon D. Tanchoco Medical Foundation (FDTMF) College of Medicine, Manila, PHL; 4 Internal Medicine, Manila Central University-Filemon D. Tanchoco Medical Foundation (FDTMF), Manila, PHL; 5 Public Health, Monroe College, New Rochelle, USA

**Keywords:** post-ercp pancreatitis, symptomatic status, asymptomatic choledocholithiasis, choledocholithiasis, endoscopic retrograde cholangio-pancreatography, diagnostic and therapeutic ercp

## Abstract

Choledocholithiasis presents variably, with some patients remaining asymptomatic, complicating decisions regarding the timing and necessity of endoscopic retrograde cholangiopancreatography (ERCP). This study represents the first meta-analysis assessing the impact of symptomatic status on post-ERCP complications and provides critical data to optimize treatment strategies. A systematic review and meta-analysis were conducted by searching PubMed, Embase, and Google Scholar through February 2024, focusing on comparing ERCP outcomes between symptomatic and asymptomatic patients with choledocholithiasis. Seven studies were included from an initial pool of 1,200 articles screened. The analysis revealed that asymptomatic patients exhibited a significantly higher overall complication rate (17.4% vs. 6.6%), including a threefold increase in the risk of developing complications overall (OR: 3.02; 95% CI: 2.26-4.03) and specifically post-ERCP pancreatitis (OR: 3.62; 95% CI: 2.63-4.99). Perforation and procedural durations were also notably higher among asymptomatic individuals. Subgroup analyses highlighted prolonged cannulation times and the use of precut sphincterotomy as potential influential factors. These findings challenge the current practice that does not differentiate based on symptomatic status and suggest a need for more tailored approaches in managing asymptomatic individuals to minimize risks associated with ERCP.

## Introduction and background

The prevalence of choledocholithiasis in the adult population of the United States is estimated at 12%, underscoring its significance in clinical gastroenterology [1]. This condition is associated with a spectrum of potential complications, including cholangitis, pancreatitis, and biliary malignancies such as cholangiocarcinoma, highlighting the critical need for effective management strategies [2]. Endoscopic retrograde cholangiopancreatography (ERCP) is currently endorsed as the primary therapeutic modality for choledocholithiasis by prevailing multi-society guidelines, reflecting its central role in the clinical management of this condition [3-6]. Interestingly, a proportion of individuals with choledocholithiasis remains asymptomatic, with estimates suggesting that up to 19% of cases do not manifest clinically evident symptoms [7]. This variability in clinical presentation complicates the decision-making process regarding the necessity and timing of intervention. While some studies have reported comparable incidences of ERCP-related complications between symptomatic and asymptomatic patients [8], others have indicated a higher complication rate among those without symptomatic disease [9], suggesting a nuanced relationship between symptomatic status and post-ERCP outcomes. Given the limited insights currently available on this topic, this systematic review and meta-analysis seek to evaluate the influence of symptomatic status on the rate of post-ERCP complications in patients with choledocholithiasis. To the best of our knowledge, this represents the first comprehensive effort to synthesize existing data on this issue, thereby aiming to contribute valuable insights to the field of Gastroenterology.

## Review

Methods

Literature Search

A systematic literature search was conducted across three major databases: PubMed, Embase, and Google Scholar. The last search was performed on February 25th, 2024. The Google Scholar search was particularly focused on the first 1000 results to maintain relevancy and manageability. The strategy aimed to identify all pertinent studies on the impact of symptomatic status on ERCP complications in patients with choledocholithiasis. We employed specific keywords and combinations including "ERCP", "choledocholithiasis", "symptomatic status", “asymptomatic”, “symptomatic”, “bleeding”, “perforation”, “pancreatitis”, "complications", among others separated by and/or boolean operators, to ensure a comprehensive collection of relevant literature.

Study Selection

Eligibility criteria included peer-reviewed observational studies (cohort and case-control) and randomized controlled trials (RCTs) that clearly differentiated between symptomatic and asymptomatic patient groups based on clinical presentation. The intervention was ERCP for diagnosing or treating choledocholithiasis. The studies required to compare outcomes between symptomatic and asymptomatic patient groups. The primary outcomes of interest encompassed complications arising post-ERCP, including pancreatitis, cholangitis, hemorrhage or bleeding, and perforation. A detailed subgroup analysis, considering variables such as patient demographic characteristics, stone, and procedural specifics, was conducted to identify any significant associations that could explain the observed outcomes. The literature search imposed no restrictions on the publication date. To ensure an emphasis on studies of high value, the study excluded non-English language studies, letters, editorials, and opinion pieces, as well as case series or reports involving fewer than 10 patients.

Screening

Two independent reviewers performed the screening process, initiating with title and abstract screening, followed by a full-text review to assess study eligibility in accordance with the inclusion and exclusion criteria. Any discrepancies between reviewers were resolved through consensus or, if necessary, discussion with a third reviewer. EndNote (Clarivate, London, United Kingdom) was used for reference management and the elimination of duplicates, while Publish or Perish software was instrumental in extracting the first 1000 relevant references from Google Scholar.

Data Extraction

Data extraction was undertaken by two independent reviewers, compiling information from included studies on variables such as participant demographic details, stone and procedural specifics, and post-procedure complications, including pancreatitis, bleeding, perforation, cholangitis, and overall complications.

Quality Assessment

The assessment of study quality and risk of bias was carried out using the Newcastle-Ottawa Scale for observational studies. Each study underwent independent assessment by two reviewers, with any disagreements resolved through discussion with a third reviewer.

Statistical Analysis

A meta-analysis was performed using RevMan Web (Cochrane, London, United Kingdom). A random-effects model was employed to accommodate potential heterogeneity across studies. Results were presented as Forest plots, summarizing effect sizes and confidence intervals for the outcomes. Heterogeneity was quantified using the I^2^ statistic, with values of 25%, 50%, and 75% indicative of low, moderate, and high heterogeneity, respectively. Sensitivity analyses were conducted to examine the impact of risk of bias on the overall findings.

Results

Search Outcomes

**Figure 1:**
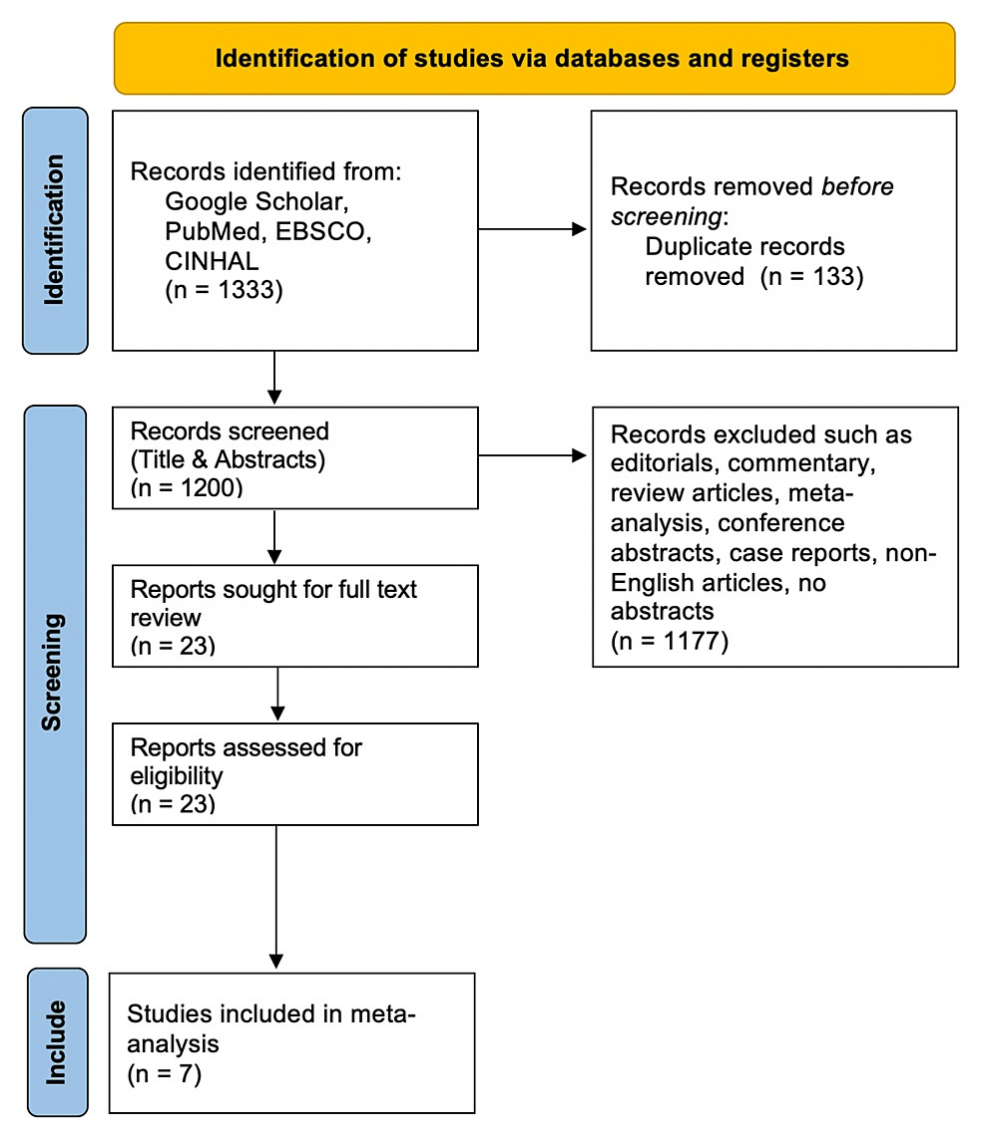
Preferred Reporting Items for Systematic Reviews and Meta-Analyses (PRISMA) flow chart

The initial comprehensive search identified 1,333 articles deemed relevant. Following the removal of 133 duplicates, the remaining 1,200 articles were subjected to title and abstract screening. The application of exclusion criteria further narrowed this selection to 23 articles eligible for full-text review. Of these, only seven studies satisfied the inclusion criteria fully. The detailed selection process is shown in the Preferred Reporting Items for Systematic Reviews and Meta-Analyses (PRISMA) flow chart, as illustrated in Figure 1.

Participant Characteristics

The characteristics of participants included in the studies are detailed in Table 1.

**Table 1:** Characteristics of study participants

Study	Total population of asymptomatic choledocholithiasis group (n)	Total population of symptomatic choledocholithiasis group (n)	Female in asymptomatic choledocholithiasis group (%)	Female in symptomatics choledocholithiasis group (%)	Age of asymptomatic choledocholithiasis group	Age of symptomatics choledocholithiasis group
Xu et al., 2020 [10]	53	274	50.9%	48.9%	49.1% age <60 in asymptomatic;	51.1% age <60 in symptomatics
Xiao et al., 2021 [11]	79	795	64.6%	51.8%	62.6±14.7 years	59.6±16.7 years
Saito et al., 2019 [12]	164	949	45.1%	46.6%	72.7±11.2 years	73.8±14.6 years
Saito et al., 2017 [9]	67	358	44.8%	47.8%	49.3% age <75 years	39.7% age <75 years
Kim et al., 2016 [13]	32	536	34.4%	45.7%	69.4±11.2 years	68.6±15.2 years
Kadokura et al., 2021 [14]	32	270	34.4%	44.8%	0% age <55	13.3% age <55
Yamaji et al., 2022 [15]	53	247	66%	61%	70.2±11.2 years	73.8±12.5 years

Risk of Bias Evaluation

The evaluation of the Newcastle-Ottawa Scale is summarized in Table 2. All included studies demonstrated high overall quality, with scores ranging from 7 to 9 points. Specifically, these studies consistently excelled in the selection domain, indicating robust participant selection methods. However, variability in scores within the comparability domain underscored challenges associated with matching groups and adjusting for confounders. Generally, outcomes were measured effectively across the studies.

**Table 2:** Risk of bias assessment with the Newcastle-Ottawa Scale

	Xu et al., 2020 [10]	Xiao et al., 2021 [11]	Saito et al., 2019 [12]	Saito et al., 2017 [9]	Kim et al., 2016 [13]	Kadokura et al., 2021 [14]	Yamaji et al., 2022 [15]
Selection	4	4	4	4	4	4	4
Comperability	1	2	2	1	2	1	2
Outcome	3	2	3	2	2	2	2
Total Score	8	8	9	7	8	7	8

Post-ERCP Complications

**Figure 2:**
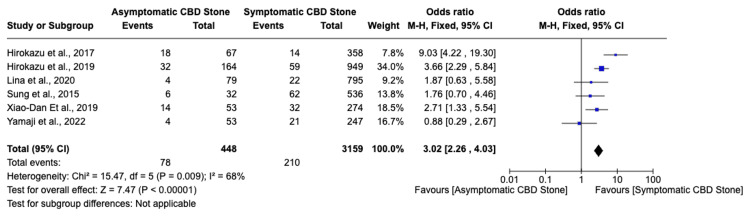
Overall complications CBD: Common bile duct

**Figure 3:**
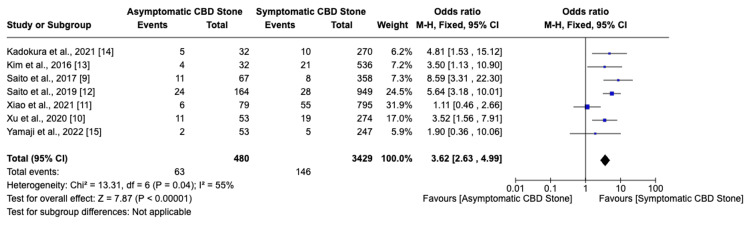
Post-ERCP pancreatitis ERCP: Endoscopic retrograde cholangiopancreatography; CBD: common bile duct

**Figure 4:**
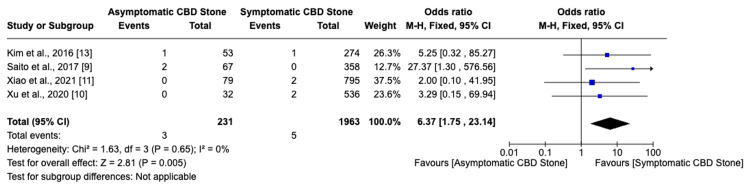
Perforation CBD: Common bile duct

**Figure 5:**
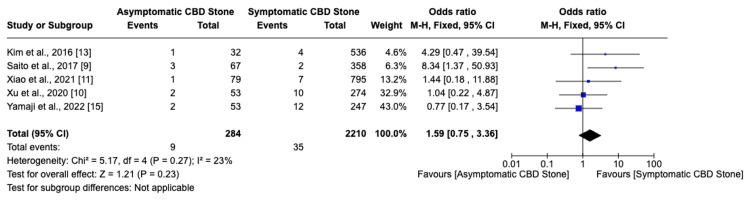
Post-ERCP cholangitis ERCP: Endoscopic retrograde cholangiopancreatography; CBD: common bile duct

**Figure 6:**
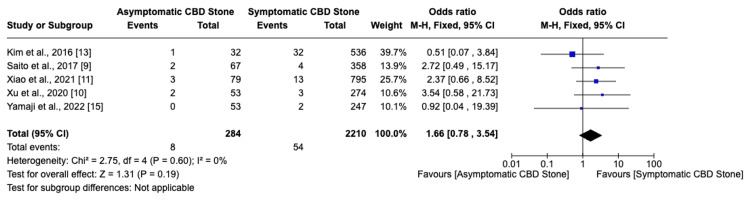
Post-sphincterotomy bleeding CBD: Common bile duct

Among seven studies, six indicated that asymptomatic choledocholithiasis patients experienced a 17.4% complication rate, compared to a 6.6% rate in symptomatic patients (Figure 2). This analysis revealed a significantly higher, threefold increase in the likelihood of developing complications in individuals with asymptomatic choledocholithiasis (OR: 3.02, 95% CI: 2.26-4.03, I²=68%). Additionally, the incidence of post-ERCP pancreatitis was 20% in the asymptomatic group versus 8.5% in the symptomatic group, indicating a similarly increased risk (OR: 3.62, 95% CI: 2.63-4.99, I²=55%) for asymptomatic individuals (Figure 3). Furthermore, the probability of perforation during or after ERCP was substantially higher in asymptomatic patients (OR: 6.37, 95% CI: 1.75-23.14, I²=0%), though the overall low incidence of this outcome warrants careful interpretation of its clinical significance (Figure 4). Studies on cholangitis reported a 3.2% occurrence rate in asymptomatic patients, nearly double that of symptomatic patients (1.6%), but without reaching statistical significance (OR: 1.59, 95% CI: 0.75-3.36, I²=23%) (Figure 5). Furthermore, the rate of post-sphincterotomy bleeding was slightly higher in the asymptomatic group (2.8% versus 2.4%), but this difference also lacked statistical significance (OR: 1.66, 95% CI: 0.78-3.54, I²=0%) (Figure 6).

Potential Factors Affecting Patient Outcomes

**Figure 7:**
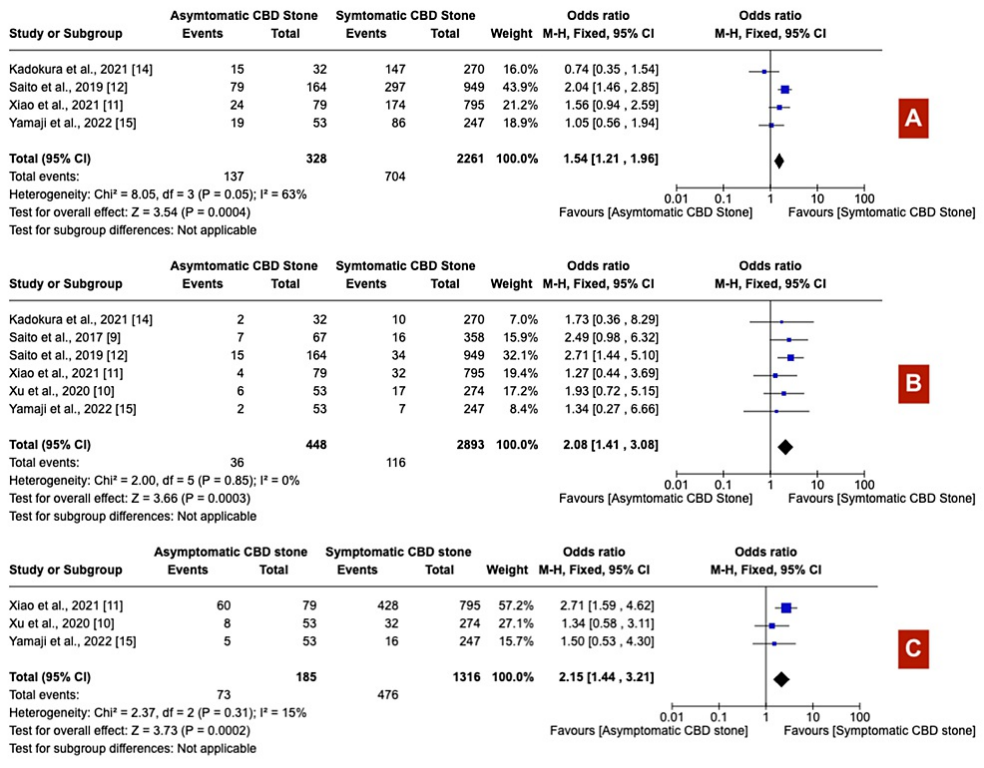
(A) Prolonged procedure time >30 mins, (B) pre-cut sphincterotomy, and (C) history of cholecystectomy CBD: Common bile duct

**Figure 8:**
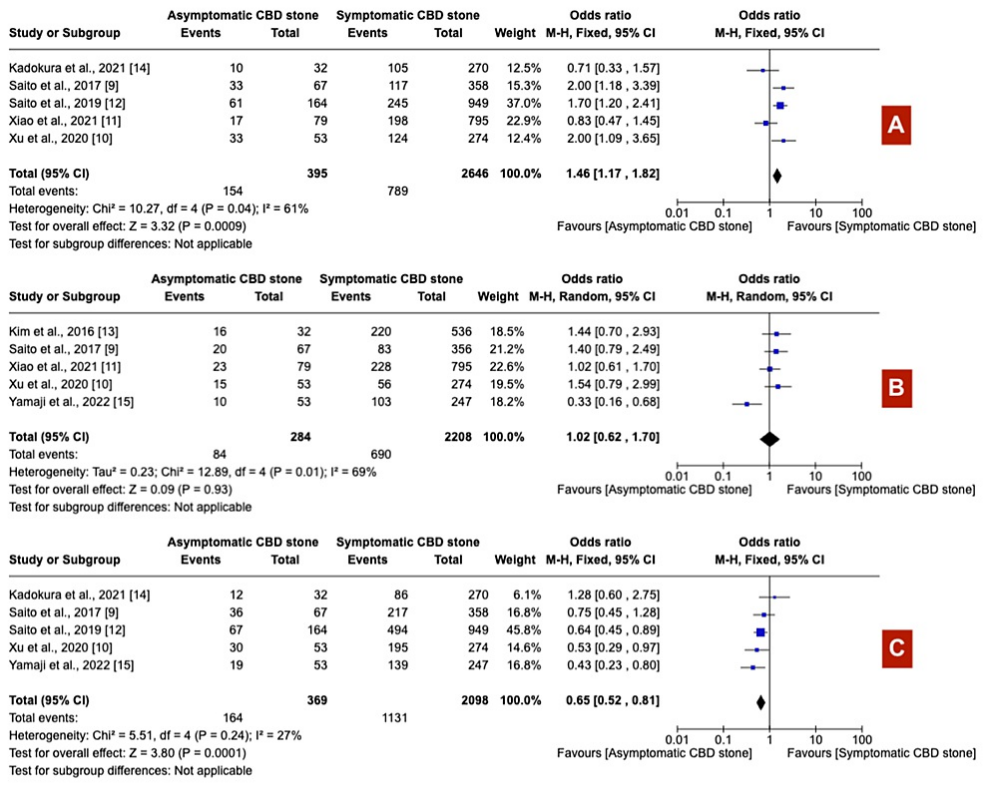
(A) Prolonged cannulation time >10 mins, (B) peri-ampullary diverticula, and (C) dilated common bile duct CBD: Common bile duct

**Figure 9:**
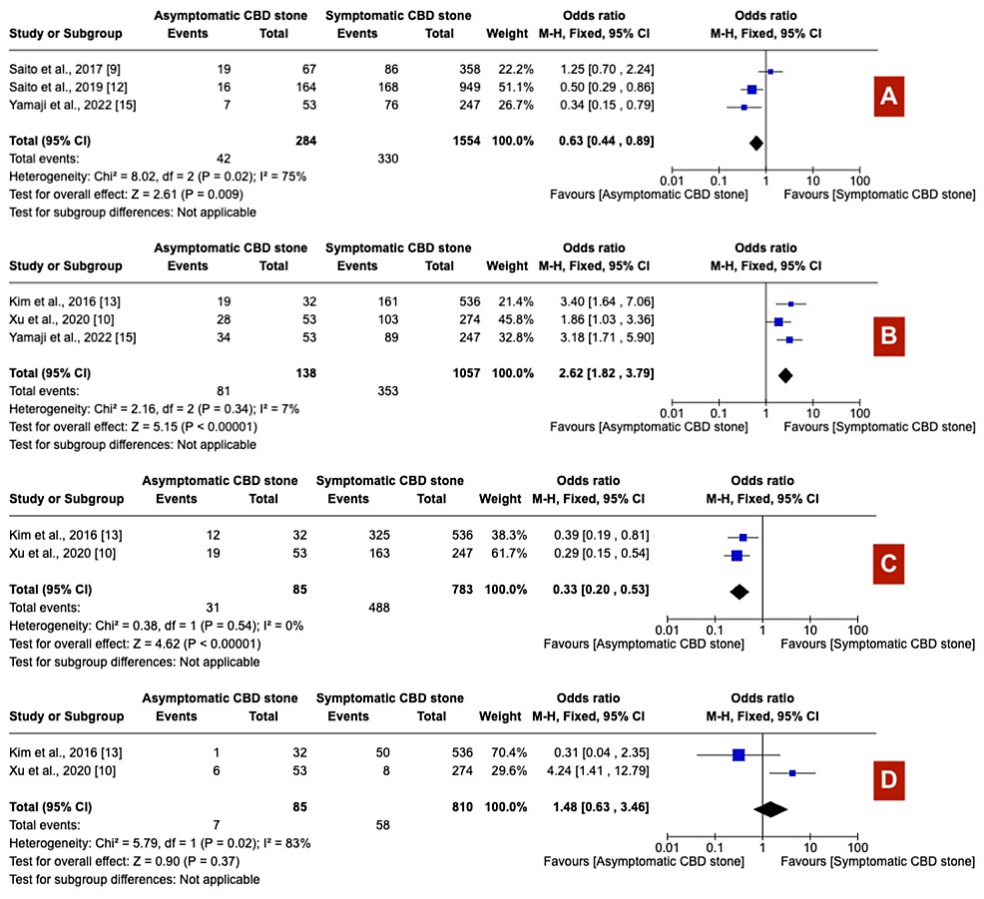
(A) Large CBD calculi, (B) black CBD calculi, (C) brown CBD calculi, and (D) cholesterol CBD calculi CBD: Common bile duct

Patients with asymptomatic choledocholithiasis underwent longer procedures (exceeding 30 minutes) more frequently (OR 1.54; 95% CI: 1.21-1.96) (Figure 7A), experienced higher rates of precut sphincterotomy (OR 2.08; 95% CI: 1.41-3.08) (Figure 7B), and reported a more frequent history of cholecystectomy (OR 2.15; 95% CI: 1.44-3.21) (Figure 7C) than their symptomatic counterparts. Furthermore, asymptomatic patients were more likely to require over 10 minutes for successful cannulation (OR 1.46; 95% CI: 1.17-1.82) (Figure 8A). The prevalence of periampullary diverticula did not significantly differ between the two groups (OR 1.02; 95% CI: 0.62-1.70) (Figure 8B). In contrast, patients with symptoms demonstrated significantly dilated common bile duct (CBD) (OR 0.65; 95% CI: 0.52-0.81) (Figure 8C) and larger calculi (OR 0.63; 95% CI: 0.44-0.89) (Figure 9A) compared to those without symptoms. Stone composition varied notably, with black calculi being more prevalent in asymptomatic patients (OR 2.62; 95% CI: 1.82-3.79) (Figure 9B), while brown calculi were more common in symptomatic individuals (OR 0.33; 95% CI: 0.20-0.53) (Figure 9C). Cholesterol calculi prevalence showed no significant difference between the groups (OR 1.48; 95% CI: 0.63-3.46) (Figure 9D). 

Discussion

This meta-analysis investigated the impacts of symptomatic status on the incidence of post-ERCP complications among patients diagnosed with choledocholithiasis. To our knowledge, this study is the first meta-analysis comparing post-ERCP complication rates between asymptomatic and symptomatic CBD stone carriers. Our analysis synthesized data from seven distinct studies. The findings reveal that asymptomatic patients exhibited a significantly higher risk of post-ERCP pancreatitis (OR 3.62, 95% CI: 2.63-4.99, I^2^ = 55%), an increased incidence of perforation (OR 6.37, 95% CI: 1.75-23.14, I2= 0%), and an increased overall complication rate (OR 3.02, 95% CI: 2.26-4.03, I^2^= 68%) when compared to symptomatic patients. Furthermore, additional variables such as the presence of black pigmented stones, prolonged cannulation times (exceeding 10 minutes), a history of cholecystectomy, the use of precut sphincterotomy, extended procedure durations (exceeding 30 minutes), and normal bilirubin levels were significantly more common among asymptomatic individuals. These outcomes may offer crucial insights into the mechanisms predisposing asymptomatic patients to an increased risk of complications following ERCP, highlighting the need for a tailored approach in the management of choledocholithiasis based on symptomatic status.

Post-ERCP pancreatitis (PEP) emerges as the most prevalent complication following ERCP, with a prevalence of approximately 14.7% as demonstrated in a previous meta-analysis involving over 13,000 patients [16]. Our study indicates that asymptomatic patients with CBD stones have a higher likelihood of developing PEP compared to symptomatic individuals (OR 3.62, 95% CI: 2.63-4.99, I^2^= 55%). Several factors may contribute to this increased risk among asymptomatic patients. First, this group often presents with non-dilated bile ducts and smaller papillary orifices due to the absence of cholestasis, complicating ERCP by making cannulation more challenging and increasing the probability of procedural complications [9]. This difficulty is highlighted by our findings, which demonstrate a lower incidence of dilated CBD in asymptomatic patients compared to symptomatic ones (odds ratio [OR] 0.65, 95% confidence interval [CI]: 0.52-0.81, I^2^=27%). Additionally, our results indicate significantly higher odds of prolonged cannulation times exceeding 10 minutes (OR 1.46, 95% CI: 1.17-1.82, I^2^=61%) and extended procedure times beyond 30 minutes (OR 1.54, 95% CI: 1.21-1.96, I^2^=63%), potentially elucidating the increased incidence of PEP in this cohort. Moreover, our analysis indicates a significantly higher prevalence of black pigment stones in the asymptomatic group (OR 2.62, 95% CI: 1.82-3.79, I^2^=7%). These stones predominantly originate from the gallbladder and are presumably smaller in size, facilitating their migration through the cystic duct to the CBD without causing symptoms or biliary infections [17]. This suggests that the size and origin of the stones might contribute to the asymptomatic presentation and the differential risk of PEP observed. However, this conclusion is derived from the analysis of only three studies, highlighting a significant limitation of our study and underscoring the necessity for investigation to fully comprehend its clinical implications and the association between stone type and the increased incidence of PEP in asymptomatic patients.

Other known risk factors associated with elevated post-ERCP complications, including pancreatitis, are precut sphincterotomy and normal bilirubin levels [18-22]. Precut sphincterotomy, involving thermal stimulation, may exacerbate edema formation in the duodenal papilla. This condition could stem from difficult biliary cannulation, thereby increasing the risk of PEP [23]. Our analysis indicates that asymptomatic patients exhibit higher odds of undergoing precut sphincterotomy compared to their symptomatic counterparts, with an OR of 2.08 (95% CI: 1.41-3.08, I^2^=0%). Furthermore, existing literature supports that a normal serum bilirubin level independently doubles the risk of PEP [22]. In our study, asymptomatic patients with CBD stones presented a significantly higher proportion of normal bilirubin levels compared to symptomatic patients (OR 20.98, 95% CI: 14.57-30.2, I^2^=75%).

Limitations of our meta-analysis include the fact that all studies included were single-center studies, highlighting the need for multicenter studies. Additionally, our study found that the post-ERCP perforation ratio was significantly higher among patients with asymptomatic choledocholithiasis compared to those with symptoms. However, the event rate was too small to draw definitive conclusions about this finding. Also, no RCTs have been conducted on this topic, guiding future studies in this area.

In summary, patients with asymptomatic CBD stones possess several risk factors that potentially increase their susceptibility to post-ERCP complications, including pancreatitis and perforations. These factors include normal serum bilirubin levels, a non-dilated CBD, prolonged cannulation times, and extended durations of the procedure. Given the association between these risk factors and the increased likelihood of complications, careful consideration is warranted when performing ERCP in individuals with asymptomatic CBD stones. Notably, current guidelines advocate for the use of ERCP in the management of choledocholithiasis, irrespective of symptomatology [4,17,24]. Consequently, despite recommendations from various national guidelines advocating for interventions in patients with asymptomatic CBD stones, a cautious approach is imperative to mitigate the risk of adverse outcomes associated with ERCP in this patient cohort.

## Conclusions

This meta-analysis represents a pioneering effort to assess the impact of symptomatic status on the frequency of post-ERCP complications in patients with choledocholithiasis. The findings indicate that patients with asymptomatic choledocholithiasis are at a higher risk of complications, including post-ERCP pancreatitis and perforation. Contributing factors may include non-dilated bile ducts, prolonged cannulation times, and the presence of black pigmented stones. These results underscore the necessity for a tailored management approach for these patients, highlighting the importance of meticulous procedural planning and the consideration of individual risk factors.
